# Case report: Two unique cases of co-existing primary brain tumors of glial origin in opposite hemispheres

**DOI:** 10.3389/fonc.2022.1018840

**Published:** 2022-12-07

**Authors:** Ishan Singhal, Dylan Coss, Wade Mueller, Michael Straza, Max Ostrinsky Krucoff, Fernando Santos-Pinheiro

**Affiliations:** ^1^ Department of Neurosurgery, Medical College of Wisconsin, Milwaukee, WI, United States; ^2^ Department of Pathology, Medical College of Wisconsin & Froedtert Hospital, Milwaukee, WI, United States; ^3^ Department of Neurosurgery, Medical College of Wisconsin & Froedtert Hospital, Milwaukee, WI, United States; ^4^ Department of Radiation Oncology, Medical College of Wisconsin & Froedtert Hospital, Milwaukee, WI, United States; ^5^ Department of Biomedical Engineering, Marquette University & Medical College of Wisconsin Graduate School, Milwaukee, WI, United States; ^6^ Department of Neurology, Medical College of Wisconsin & Froedtert Hospital, Milwaukee, WI, United States

**Keywords:** neuro-oncology, oligodendroglioma, glioma, astrocytoma, co-existing tumors

## Abstract

**Background:**

Primary CNS tumors are rare. Coexistence of two glial tumors of different histological origins in the same patient is even rarer. Here we describe two unique cases of coexisting distinct glial tumors in opposite hemispheres.

**Cases:**

Patient 1 is a 38-year-old male who presented with a seizure in February/2016. MRI showed a left parietal and a right frontal infiltrating nonenhancing lesions. Both lesions were resected revealing an oligodendroglioma WHO grade-2 and an astrocytoma WHO grade-2. Patient 2 is a 34-year-old male who presented with a seizure in November/2021. MRI showed a left frontal and a right mesial temporal lobe infiltrating nonenhancing lesions. Both lesions were resected revealing an oligodendroglioma WHO grade-2 and a diffuse low-grade glioma, MAPK pathway-altered (BRAF V600E-mutant). Patient 1 underwent adjuvant treatment. Both patients are without recurrence to date.

**Discussion:**

Two histologically distinct glial tumors may coexist, especially when they are non-contiguous. Pathological confirmation of each lesion is imperative for appropriate management. We highlight the different management of gliomas based on the new CNS WHO 2021 classification compared to its 2016 version, based on NCCN guidelines. Although more molecular markers are being incorporated into glioma classification, their clinical impact of it is yet to be determined.

## Introduction

Primary Central Nervous System (CNS) tumors are rare and account for about 2% of all cancers with an overall incidence of 22 per 100,000 population (1). Glioma is the most common type of primary brain tumor, with astrocytoma accounting for about half of these cases while oligodendrogliomas account for around 5% of primary intracranial tumors (2). While primary brain tumors are rare, the co-existence of gliomas of different histologic and molecular categories in the same individual in opposite hemispheres has never been reported in the literature to our knowledge. A total of 72 total cases of coexisting histologically distinct primary CNS tumors have been reported, most of them describing the coexistence of meningiomas and gliomas. None describe coexisting glial tumors from distinct histologic and molecular categories (3–29). Here we report two unique cases of coexisting glial tumors with distinct histologic and molecular profiles, located in opposite brain hemispheres. It is important to acknowledge the presence of this phenomenon, as otherwise these disparate lesions may be falsely assumed to be multifocal pathology and/or gliomatosis as opposed to two separate low-grade lesions.

## Case series

### Patient 1

**Figure 1:**
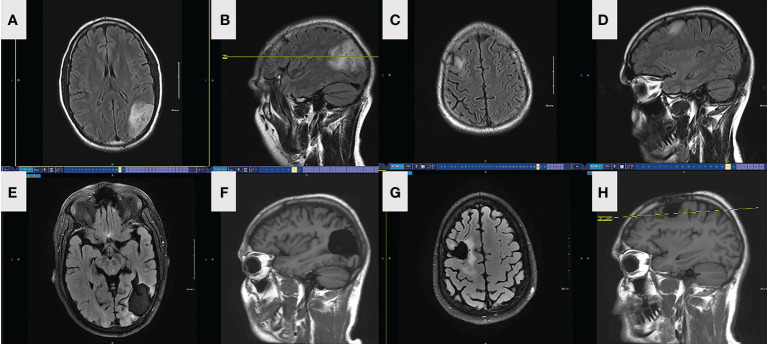
FLAIR axial **(A)** and sagittal **(B)** MRI depicting preop left parietal lesion in patient 1. FLAIR axial **(C)** and sagittal **(D)** MRI depicting preop right frontal lesion in patient 1. FLAIR axial **(E)** and sagittal **(F)** MRI depicting left parietal lesion and resection in patient 1, 18-month postoperatively. FLAIR axial **(G)** and sagittal **(H)** MRI depicting right frontal lesion and resection in patient 1, 18-month postoperatively.

A 38-year-old male presented to the ED after experiencing a new onset GTC seizure in February 2018. MRI brain was eventually obtained revealing two infiltrating, expansile, and non-contiguous T2-FLAIR hyperintense lesions on the left parietal and right frontal lobes. Faint enhancement was seen in the posterior aspect of the left temporal lesion (**Figure 1**). MR Spectroscopy revealed elevated choline to creatine and choline to N-acetylaspartate (NAA) ratios in the left temporoparietal lesion, suggestive of a neoplastic process. The right frontal lesion was too small and close to the skull and therefore not tested. Preoperative CT did not show any intralesional calcifications.

**Figure 2:**
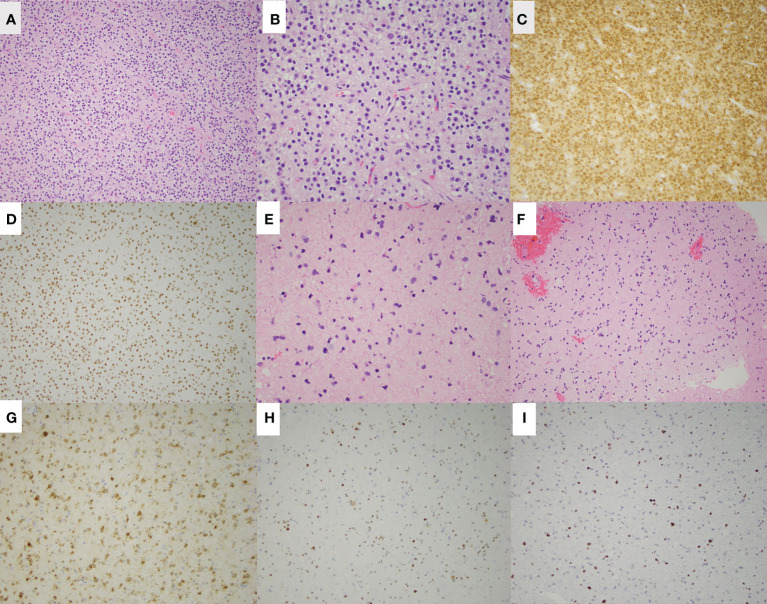
H&E staining of the left parietal oligodendroglioma in patient 1 is shown at low magnification **(A)** and high magnification **(B)**. Additional staining of the left parietal lesion shows IDH-1 (R132H) mutation **(C)** and ATRX retained **(D)**. H&E staining of the right frontal astrocytoma is shown at low magnification **(E)** and high magnification **(F)**. Additional staining shows IDH-1 (R132H) mutation **(G)**, ATRX loss **(H)**, and p53 overexpression **(I)** of the right frontal astrocytoma.

He underwent a craniotomy and GTR of the left parietal lesion on 6/2018. Pathology revealed oligodendroglioma, WHO grade 2, IDH-mutant, 1p/19q-codeleted. One month later, he underwent a second craniotomy and GTR of the right frontal lesion. Pathology revealed an astrocytoma, WHO grade 2, IDH-mutant, ATRX-mutant, and 1p/19q-intact (**Figure 2**). Intraoperative awake electrocorticography recorded brief, non-evolving focal epileptic activity described as 5-10 low-amplitude periodic spikes arising from the left temporoparietal region.

The patient did well postoperatively and remains seizure-free while on antiepileptic monotherapy. He developed chronic right homonymous hemianopsia but is otherwise cognitively and physically intact. The decision was made to treat the astrocytic right frontal lesion with intensity-modulated radiation therapy (IMRT) (total of 60Gy in 30 fractions) followed by temozolomide for a total of 12 cycles to treat both pathologies. He is currently doing well without recurrence or worsening neurological symptoms at his last follow-up on 1/11/2022. He continues to work three jobs and is excited about getting married in February.

### Patient 2

**Figure 3:**
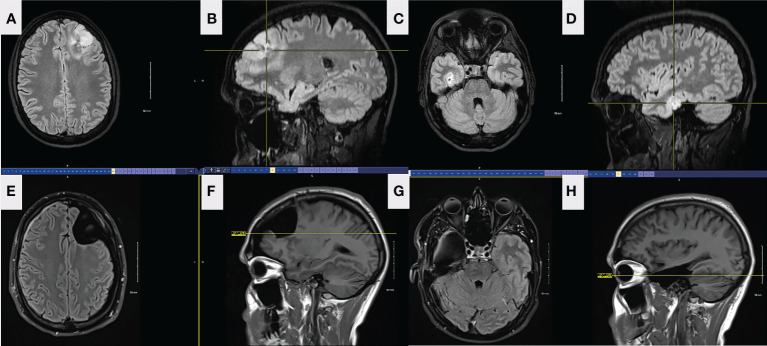
FLAIR axial **(A)** and sagittal **(B)** preop MRI of the left frontal lesion in patient 2. FLAIR axial **(C)** and sagittal **(D)** preop MRI of the right temporal lesion in patient 2. Axial **(E)** and sagittal **(F)** FLAIR MRI from left frontal tumor resection in patient 2, 5 months postoperatively. The resection was taken around all margins of the FLAIR lesion until functional borders were found, including into the corpus callosum across the midline. FLAIR MRI axial **(G)** and sagittal **(H)** from right temporal tumor resection in patient 2, 4 months postoperatively. The resection was taken beyond the margins of the FLAIR lesion.

A 34-year-old right-handed male presented to the emergency department (ED) in September 2021 after new-onset episodes of expressive aphasia, each lasting less than 2 minutes. While in the ED, he developed two generalized tonic-clonic (GTC) seizures. MRI brain demonstrated two expansile, non-enhancing, and non-contiguous T2-FLAIR hyperintense lesions on the left frontal and right mesial temporal lobes (**Figure 3**). Preoperative CT showed gyriform and ill-defined calcification associated with the left frontal lobe mass, consistent with oligodendroglioma. There were no calcifications in the right temporal mass. The patient was admitted to the hospital, started on dexamethasone and levetiracetam, and prepped for surgery.

He underwent an uncomplicated awake craniotomy and gross total resection (GTR) of the left frontal lesion with intraoperative MRI. Pathology was consistent with oligodendroglioma, WHO grade 2, IDH-mutant, 1p/19q-codeleted.

**Figure 4:**
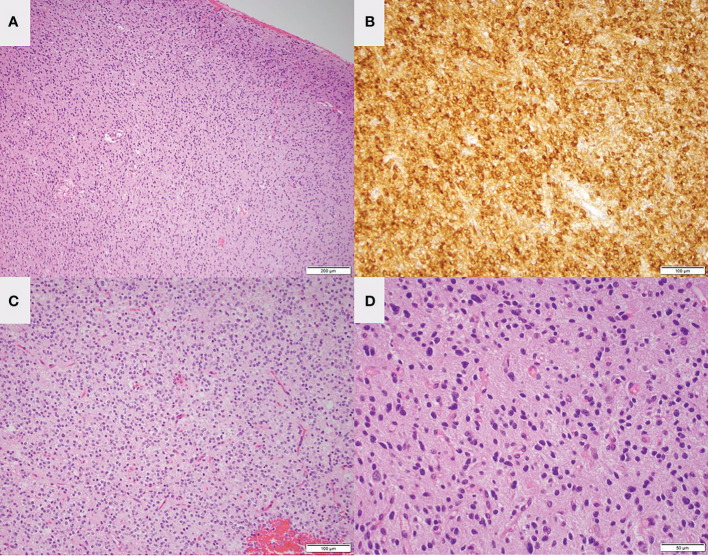
Low magnification H&E staining **(A)** and IDH mutant staining **(B)** of the left frontal lesion in patient 1 are consistent with oligodendroglioma. Low magnification **(C)** and high magnification **(D)** H&E staining of the right temporal lesion is consistent with low-grade glioma.

After he recovered from the first surgery, he underwent a right temporal craniotomy and subtotal resection of the contralateral lesion on the right mesial temporal lobe. Morphologic and molecular findings from this specimen were most suggestive of diffuse low-grade glioma, MAPK pathway altered as the tumor showed evidence of BRAF mutation (V600E). The tumor is notably IDH wildtype, H3 wildtype, 1p/19q not co-deleted, and no evidence of CDKN2A deletion, indicating a favorable prognosis (**Figure 4**) (30). A current WHO grade is yet to be assigned for these neoplasms according to the recent 2021 WHO guidelines (31). Intraoperative awake electrocorticography did not record any epileptiform activity.

The patient did well after both surgeries and remained seizure-free while on monotherapy antiepileptic. He remained both clinically and radiographically stable at his 6-month follow-up visit. He is currently doing well without recurrence; the most recent follow-up was on 6/7/2022. He works full-time as an engineer but has transitioned to an office job as he had trouble with extreme temperatures. He feels back to his baseline with infrequent memory problems.

## Discussion

Here we describe two unique cases of co-existing glial tumors from distinct cellular lineages on opposite brain hemispheres. Both patients initially presented following new-onset seizures. They both underwent two separate craniotomies for maximal safe resection of each of the lesions. Both patients had coexistence of oligodendroglial and an astrocytic tumor, one in each hemisphere.

To our knowledge, this is the first report of co-existing glial tumors from distinct histologic and molecule profiles on opposite brain hemispheres. It is critical to recognize this as part of the differential diagnosis when evaluating patients with two discrete, non-contiguous lesions, as historically (i.e., prior to the era of molecular markers) these may have been misdiagnosed as gliomatosis and/or multifocal pathologies. A literature review conducted by Tunthanathip et al. identified 6 cases of coexisting intracranial tumors and summarized the author’s experiences. They then identified 65 similar cases described in the literature. They noted that the most common association was a meningioma and pituitary adenoma, and that meningioma was present in most of their reported cases (32). Additionally, Nalmada et al. describe a case report of co-existing astrocytoma and oligodendroglioma both in the right frontal lobe (33).

The strikingly different mutation and methylation profiles observed in the two tumors in each case (**Figures 2**, **4**) suggest that each neoplasm had distinct tumorigenesis and even place of origin, considering their location in opposite hemispheres. Additionally, no genetic mutations have been identified to suggest a cancer-predisposition syndrome or familial hereditary syndrome in either case. Neither patient had a personal or family history suggestive of genetic predisposition, and therefore no genetic counseling was done.

Another important point is the impact of the new 2021WHO classifications on management when compared to the 2016 WHO classification (31, 34). Prior to this update, Patient #2’s temporal tumor would have been classified as diffuse astrocytoma, IDH-wildtype, WHO grade II according to the WHO 2016 classification, and therefore would have been treated with adjuvant radiation and chemotherapy at our institution. This use of adjuvant radiation and chemotherapy is highlighted in the treatment of patient #1’s right frontal astrocytoma three years earlier. With the most recent 2021 WHO classification, the tumor is now classified as low-grade glioma, MAPK pathway altered, due to the tumor’s molecular profile. This tumor carries a lower risk of recurrence and does not have a WHO number (2,3 or 4) associated with it; given his age, radiographic surveillance without adjuvant therapy was recommended (35).

This manuscript describes two rare cases of coexisting glial tumors in opposite brain hemispheres, and it illustrates the differences in glioma classification comparing the 2021 WHO classification to its previous 2016 version. It also highlights the difference in the management of brain tumors based on an integrated diagnosis strategy as recommended by NCCN guidelines.

## Data availability statement

The original contributions presented in the study are included in the article/supplementary material. Further inquiries can be directed to the corresponding author.

## Author contributions

FP and MK contributed to the conception and design of the case report. IS wrote the first draft of the manuscript. FP, MK, and IS wrote sections of the manuscript. DC provided histologic imaging. MS contributed to the acquisition of the data, its analysis, and interpretation. All authors contributed to the manuscript revision, read, and approved the submitted version.

## Conflict of interest

The authors declare that the research was conducted in the absence of any commercial or financial relationships that could be construed as a potential conflict of interest.

## Publisher’s note

All claims expressed in this article are solely those of the authors and do not necessarily represent those of their affiliated organizations, or those of the publisher, the editors and the reviewers. Any product that may be evaluated in this article, or claim that may be made by its manufacturer, is not guaranteed or endorsed by the publisher.
